# Ejaculate Allocation and Sperm Characteristics Differ among Alternative Male Types in a Species of Fish with Cooperation and Competition among Unrelated Males

**DOI:** 10.3390/cells10102612

**Published:** 2021-10-01

**Authors:** Suzanne H. Alonzo, Kelly A. Stiver, Holly K. Kindsvater, Susan E. Marsh-Rollo, Bridget Nugent, Erem Kazancıoğlu

**Affiliations:** 1 Department of Ecology and Evolutionary Biology, Institute of Marine Sciences, University of California Santa Cruz, 130 McAllister Way, Santa Cruz, CA 95060, USA; 2 Department of Psychology, Southern Connecticut State University, 501 Crescent Street, New Haven, CT 06515, USA; stiverk1@southernct.edu; 3 Department of Fish and Wildlife Conservation, Virginia Polytechnic Institute and State University, Blacksburg, VA 24061, USA; hkindsvater@vt.edu; 4 Department of Psychology, Neuroscience and Behaviour, McMaster University, 1280 Main St. W, Hamilton, ON L8S 4K1, Canada; marshse@mcmaster.ca; 5 Department of Ecology and Evolutionary Biology, Yale University, New Haven, CT 06515, USA; brie423@gmail.com; 6 Protenus, Inc., 1629 Thames St., Baltimore, MD 21231, USA; erem.kazancioglu@gmail.com

**Keywords:** sexual selection, sperm competition, ejaculate evolution, alternative reproductive types, reproductive strategies, phenotypic plasticity, *Symphodus ocellatus*, Labridae

## Abstract

Sexual selection arising from sperm competition has driven the evolution of immense variation in ejaculate allocation and sperm characteristics not only among species, but also among males within a species. One question that has received little attention is how cooperation among males affects these patterns. Here we ask how male alternative reproductive types differ in testes size, ejaculate production, and sperm morphology in the ocellated wrasse, a marine fish in which unrelated males cooperate and compete during reproduction. Nesting males build nests, court females and provide care. Sneaker males only “sneak” spawn, while satellite males sneak, but also help by chasing away sneakers. We found that satellite males have larger absolute testes than either sneakers or nesting males, despite their cooperative role. Nesting males invested relatively less in testes than either sneakers or satellites. Though sneakers produced smaller ejaculates than either satellite or nesting males, we found no difference among male types in either sperm cell concentration or sperm number, implying sneakers may produce less seminal fluid. Sperm tail length did not differ significantly among male types, but sneaker sperm cells had significantly larger heads than either satellite or nesting male sperm, consistent with past research showing sneakers produce slower sperm. Our results highlight that social interactions among males can influence sperm and ejaculate production.

## 1. Introduction

Sperm competition drives sexual selection on a variety of male traits, including the amount of energy males allocate to sperm production, the type of sperm they produce, and the quantity of and compounds found in their seminal fluid [1,2,3,4,5,6,7,8,9]. In fact, sperm cells are one of the most diverse and rapidly evolving cells in the animal kingdom [10,11], and divergence in seminal fluid proteins contributes to reproductive isolation and therefore speciation and biodiversity [12,13,14]. Not only do sperm and other ejaculate traits diverge among populations and species [4,9,15,16,17,18], these traits also differ among individuals in the same population [3,19,20,21,22,23], and even change over the lifetime of an individual in response to their experience, condition, and social environment [24,25,26,27,28,29,30]. All else being equal, sperm velocity, motility and longevity are expected to be higher in species with more frequent and intense sperm competition [31,32] and for individuals within species that experience chronically higher sperm competition [1,2,5]. Releasing more sperm and producing faster sperm also generally increases fertilization success [32,33]; but see [34].

One powerful approach for studying how sexual selection arising due to sperm competition has shaped sperm characteristics and ejaculate production has been to compare alternative male reproductive types that differ in the risk or intensity of sperm competition they experience. We use the general term “alternative male types” (rather than alternative tactics or strategies) to imply the coexistence of multiple male phenotypes that differ in reproductive traits (without implying the degree to which these phenotypes are genetically determined versus plastic). Alternative male types that experience consistently higher sperm competition are expected, all else being equal, to invest more in sperm production and to produce sperm that are more likely to be successful in competition [1,2,5]. Multiple studies have found support for the prediction that alternative male types that experience chronically higher levels of sperm competition will invest more in ejaculate production [3,4,35,36]. However, reviews synthesizing how sperm traits differ among male types within species did not find that sperm traits consistently differ among alternative male types according to their risk and intensity of sperm competition [23,36]. For example, male types that experience greater sperm competition do not have consistently faster sperm, greater motility or greater longevity [23]. This highlights the need to understand more about the degree to which sperm production and traits are plastic, and how individual experience and social interactions affect patterns of sperm production [19,23]. Studying how sperm characteristics and production differ in a species with plastic (rather than genetically determined) alternative male reproductive types allows us to explore these questions.

What these syntheses have shown is that extensive unexplained variation in ejaculate allocation and sperm traits remains. One interesting question that has not received much attention is how sperm production and characteristics will differ among males adopting alternative reproductive types in species where there is both reproductive cooperation and competition among males (but see [24,36,37]). More generally, it is interesting to ask whether, and if so, how, the social role that males play in the mating system and the social interactions they experience affects their sperm production, ejaculate allocation and sperm characteristics. In some species with cooperation among related or unrelated males, the “helper” males are functionally nonreproductive, meaning they have inactive or reduced testes that do not produce viable sperm [37,38]. In other species, cooperative males are reproductively capable, but have low investment in testes and sperm production, which could be a concession to dominant males in order to stay in the social group [39]. In still others, males exhibit reproductive helping or cooperative behaviors, and yet still compete actively for reproductive resources, mating opportunities and fertilizations (e.g., [19,40,41]). Yet, very few studies have asked whether, and how, the presence of cooperative and competitive interactions among unrelated males influence sperm characteristics, or sperm and seminal fluid production (but see [25,38]). In fact, we know more about cooperation among sperm cells [42,43,44,45] than how cooperation among males alters selection on sperm and ejaculates.

Here we ask how male alternative types differ in gonadal allocation, ejaculate production and sperm morphology in a species with plastic alternative reproductive types, the ocellated wrasse (*Symphodus ocellatus*, family Labridae). In these wrasses, unrelated males both compete and cooperate during mating and fertilization. Nesting males defend territories and build nests out of algae in which females lay their eggs [46,47]. Fertilization is external and smaller sneaker males hover near nests and join the nesting male and female during a spawning to release sperm and compete for fertilizations [46]. The presence of sneaker males is strongly correlated with the number of females visiting and spawning in nests [48,49]. Satellite males are intermediate in size and coloration, and tend to associate with a nest and nesting male for a few days during the spawning phase of the 7–10 day nest cycle (which involves building, spawning and parental care, [46,50]). Satellite males sneak spawn, but they also attempt to court females and bring them to the nest, and chase sneaker males away from the nest [41]. Thus, they cooperate behaviorally with their unrelated nesting male partner, reducing his risk of sperm competition as well as their own, while they also compete directly with both nesting males and sneakers for fertilizations [41,51].

The alternative male types in the ocellated wrasse are not due to a genetic polymorphism. Instead, male phenotype changes between reproductive seasons, depending on juvenile growth and body size [52]. Yet the male types differ in many aspects of behavior, physiology, gene expression, and sperm characteristics [46,50,52,53,54,55]. In this species, cooperation among males is directly related to reducing the intensity of sperm competition and is not related to parental care or territory defense [41]. The system is therefore an excellent one in which to ask how social interactions occurring at the time of mating affect sperm and ejaculate production and characteristics.

The risk and intensity of sperm competition are high for all males in the wrasse system. For the nesting male, on average, one third of spawns involve competition with other males [49,56,57]. The risk and intensity of sperm competition that an individual nesting male experiences also varies over time. Nesting males experience little or no sperm competition on some days, and high rates of sperm competition on other days, in which they compete with as many as 20 sneaker males [49,56]. Sneakers are always, and satellites are almost always, in sperm competition with the nesting male, and both are often in competition with multiple other males as well [46,50,56]. Past research found that relative testes size (i.e., the gonadosomatic index, GSI) is highest for sneaker males, intermediate for satellite males and lowest for nesting males [57], as expected based on the differences in the average risk and intensity of sperm competition they experience [1,2]. However, prior work did not report either absolute testes size or the allometric relationship between body size and testes size, which is now recognized to be a better way to compare gonadal allocation among types [58]. Alonzo and Warner [56] found that on average sneaker males release four times as many sperm for each spawning event than either satellite or nesting males. Despite these differences in GSI and the number of sperm released, sneaker and satellite males have much lower paternity than nesting males, which sire roughly two thirds of all offspring [59,60,61,62]. Sneaker males produce sperm with lower velocity than nesting males [63], while satellite male sperm has not yet been compared to the other male types. An open question is how testes size, sperm and seminal fluid production and sperm characteristics of these behaviorally cooperative satellite males compare to the same variables in the sneaker (parasitic) and nesting (dominant) male social and reproductive types.

Here, we report empirical research comparing testes size, ejaculate (sperm and seminal fluid) production, and sperm cell morphology among alternative male reproductive types (sneaker, satellite and nesting males) in the ocellated wrasse (*S. ocellatus)*. All else being equal, a greater risk and intensity of sperm competition (i.e., sneakers > satellite males > nesting males) is expected to be associated with increased allocation to testes (consistent with [57]), greater ejaculate production, more rapid ejaculate regeneration, and sperm characteristics associated with a greater ability to fertilize eggs under sperm competition (e.g., longer tails and smaller sperm cell heads). Yet, this might also change, given that satellite males not only compete for fertilizations but also cooperate with nesting males. Satellite males might therefore be expected to have smaller testes or less competitive sperm due to this cooperative role and their dependence on nesting males for access to the nest. Yet, satellite males are in competition with the nesting male, as well as sneaker males. In addition, these alternative male types differ not only in sperm competition, but also in their absolute body size, condition and energy budget. We therefore also expect that the predicted patterns based on sperm competition alone may hold on a relative scale but not necessarily also on an absolute scale. This is because males with more energy may be able to produce larger testes, more sperm, or higher quality sperm. Our primary goal is to contribute to the understanding of how sperm traits and ejaculate production (and their plasticity) evolve in response to sperm competition and social interactions in general by asking how these traits are affected by the existence of both reproductive competition and cooperation among unrelated males more specifically.

## 2. Materials and Methods

### 2.1. Study Site and General Information

This research was conducted May–June from 2009–2014 and in 2019 in the Baie de Revellata of the Mediterranean Ocean at STARESO, a marine research field station in Corsica, France. All behavioral observations and fish collections were made underwater on SCUBA in a rocky reef habitat along roughly 200 m of coastline near the field station at or above 10 m depth. Fish were caught using two small handheld nets and held in Fabrill minnow bait buckets while being transported back to the lab at the field station, where they were held in large tanks with free flowing seawater until further study, as described below. All of the statistical analyses reported in this paper were conducted in RStudio Version 1.2.5033 (Integrated Development for R. RStudio Inc., Boston, MA, USA).

### 2.2. Absolute and Relative Allocation to Gonads

From 2009–2014 and in 2019, we collected fish from actively spawning nests after a brief observation to confirm their reproductive type and status (i.e., female, sneaker, satellite or nesting male), as determined by well-documented behavioral and morphological differences among the types [41,46,49,52,56]. After capture and being brought back to the lab, these fish were euthanized using an overdose of MS-222. We then measured standard length to the nearest millimeter using dial calipers and total wet body weight to the nearest 0.01 g using a Bauhaus Scout Pro Balance. The gonads (testes or ovaries) were then dissected out and weighed using the same balance and level of precision.

Our dataset included 72 sneaker males, 54 satellite males, 84 nesting males and 68 females. For each individual, we had data on their total body weight, gonad weight, standard length and their type (i.e., female, sneaker, satellite or nesting male). From these data we also calculated soma weight (i.e., total weight−gonad weight) and condition factor (i.e., body weight/standard length^3^). We also calculated the natural log (ln) of gonad weight, soma weight and standard length for the allometric analyses. We then looked at a histogram of the gonad weight and dropped 3 outliers (2 females and one sneaker male sample) for which gonad weight was below 0.03 g because this likely represented samples in which either the dissection failed or the fish was not reproductive. This left us with data from 71 sneaker males, 54 satellite males, 84 nesting males and 66 females.

**Figure 1:**
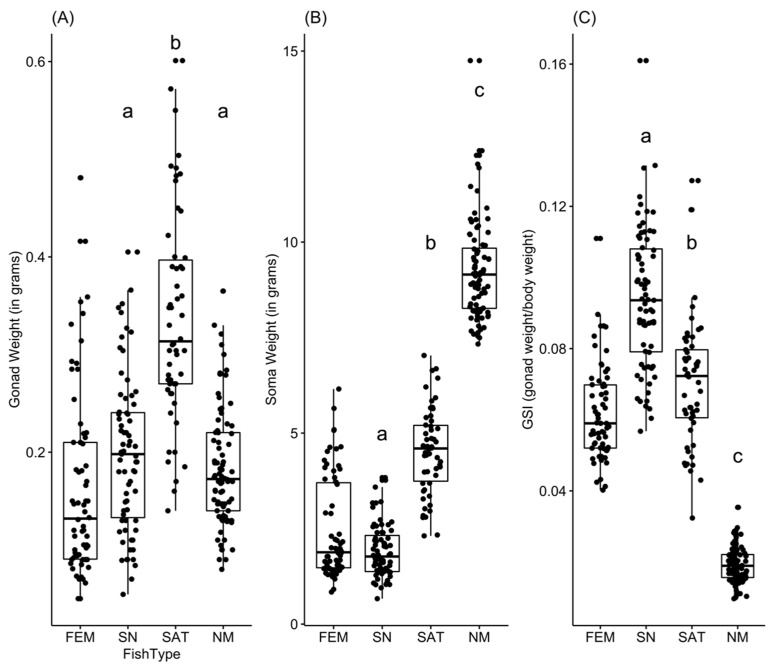
Alternative reproductive types differ in gonad weight and soma weight, as well as variation in gonad weight. On the x-axis: FEM = females, SN = sneaker males, SAT = satellite males, NM = nesting males. (**A**) Absolute gonad weight differs among types; satellite males have the largest absolute testes. (**B**) Absolute soma weight differs among types; sneakers are smaller than satellite males and satellite males are smaller than nesting males. (**C**) Gonadosomatic index differs among male types. Female data for all three variables are included for graphical comparison, but were not included in the statistical analyses. Lower case letters represent the male types that are significantly different from one another based on ANOVA followed by Tukey post hoc pairwise comparison tests. Alternative male types also differ significantly in GSI (sneaker > satellite > nesting male relative gonad weight). See text for full statistical methods and results.

Using these data, we first asked whether there were significant differences among alternative male types in absolute testes weight, soma weight and body condition (using Fulton’s body condition index = body weight/(standard length^3^). We used ANOVA followed by Tukey post hoc tests, after testing for extreme outliers (using identify_outliers in R), deviations from normality, or evidence of nonhomogeneity of variances. When deviations were found, we first attempted Box−Cox transformation of the data (using boxcoxfr in R). If the data could not be transformed to meet the assumptions of ANOVA, we conducted a Kruskal−Wallis test followed by a Dunn’s post hoc test. We also report the gonadosomatic index (GSI = gonad weight/total body weight) but did not perform our analyses on GSI, but instead estimated the allometric relationship between body size and testes size (which is recognized to be a better way to compare gonadal allocation among types [58]). To be certain our results were robust, we also repeated these analyses including females in the dataset for comparison, which did not affect the qualitative results and therefore are not reported here. We do, however, present the data on female ovary and soma weight in Figure 1 (see below) for reference and qualitative comparison between the sexes. Finally, we conducted the same analyses using standard length and body condition instead of soma weight. These analyses yielded the same qualitative results, but the model with soma weight had a greater ability to explain variation in testes weight, which we therefore focus on here. We then asked whether alternative male types differ in relative gonadal allocation by estimating the linear relationship between ln(gonad weight) and ln(soma weight) and asking whether male types differed significantly in this allometric relationship. We first attempted to use ANCOVA (using aov and lm in R) to estimate this relationship (following methods outlined in [58]). However, our dataset violated two assumptions of ANCOVA: homogeneity of regression slopes and independence of the covariate and treatment effect [64,65]. We therefore used multilevel models and fitted a mixed effects model using maximum likelihood (using lme in R) and found the best fit model using log-likelihood [65].

### 2.3. Ejaculate Production and Regeneration

In 2012, males of all three types were first observed and caught at actively spawning nests and then brought back to the lab for study, as described above. These collections took place early in the morning to ensure that we obtained samples that represented the initial volume of sperm and seminal fluid (i.e., the male’s ejaculate, also called milt in fish) available to males at the beginning of the day. Mating occurs throughout daylight hours during the breeding season. We noted the type of fish (e.g., sneaker, satellite or nesting male) as well as their standard length and wet weight. Within one hour of being caught, we collected initial ejaculate samples by having one researcher press gently on the fish’s abdomen near their genital papillae while a second person used a micropipette to collect the sample. We continued collecting until gentle pressure no longer yielded any milt. We determined the total volume of the ejaculate (sperm and seminal fluid sample, measured in microliters), activated the sample in 0.5 mL of seawater, and after a few minutes added 9 microliters formalin such that the sperm cells were preserved in ~2% formalin so the sample concentration and total number of sperm cells in the sample could be estimated later. The fish were then held in small tanks with running seawater from the same site as the fish were collected for 5 h after which we collected a second milt sample using the same methods. Fish were then released at the study site. We chose 5 h between samples based on a pilot study in which we varied the number of hours between samples and found that after shorter periods (e.g., two hours) most males had not regenerated sperm. In addition, more frequent or repeated sampling appeared stressful for the fish.

We later added a 3-microliter subsample of the preserved sperm samples to one chamber of a Leja 4-chamber depth glass slide to count sperm cells in order to estimate sperm cell concentration and total number in the sample. For each sample (two per male), we took 20 different images using a Leica DM2500 phase contrast microscope and attached digital camera at 400 times total magnification. We also took an image of a stage micrometer to determine the area of the image captured by the digital camera attached to the microscope. We counted the number of sperm in each microscope image to determine sperm cell concentration (mean sperm cells per image). These sperm counts along with the depth of the chamber (20 microns) and area of each image (0.09 mm^2^, so the sample volume captured in each image was 0.0018 μL) and the total sample volume (milt sample volume + 500 μL seawater + 9 μL formalin) were used to estimate the total number of sperm cells in the sample.

We excluded two replicates in which the first sample did not contain sperm, as this likely meant the male was either nonreproductive or something went wrong during sample collection, preservation or counting. We also tested for and dropped extreme outliers (using identify_outliers in R), if found. As described in greater detail above, we used ANOVA (when the assumptions of normality and homogeneity of variance could be met) or Kruskal−Wallis test (when they could not) to ask whether alternative male types differed in the volume, concentration or estimated sperm number in their first ejaculate (i.e., milt sample containing sperm and seminal fluid available to them at the beginning of a reproductive day). If significant differences were found, we then used either Tukey or Dunn’s post hoc tests to determine which groups differed significantly from one another. Using linear regression and model comparison, we also looked for evidence of a tradeoff between seminal fluid and sperm cell production by asking whether there was a significant linear relationship between sample volume and sperm cell concentration and whether male type had a significant effect on this relationship. We also compared the same three variables between the first and second samples for each male in a paired design. It was not possible to use either a repeated measures ANOVA or mixed effect model to determine whether males differed in the sperm and seminal fluid regeneration due to significant deviations from the assumptions of these models [66]. We therefore calculated the difference between the first and second samples for each individual for all three variables and compared among male types using ANOVA or Kruskal−Wallis tests when the assumptions of the ANOVA were not met.

### 2.4. Sperm Morphology

In 2011 and 2012, live fish were caught at actively spawning nests as described above and brought back to the lab for study. We collected and preserved sperm samples from these males using the same methods as described above. For this part of the study, however, only one initial sample was taken. As above, a 3-microliter subsample of the preserved sperm sample was added to the chamber of a Leja 4-chamber slide and images of these samples were taken to be used to measure sperm characteristics. We again took images of a standard stage micrometer to calibrate our measurements. Using ImageJ, we measured the length of the sperm tail and the area of the head of 10 sperm cells for every sample (except in one case for which only 8 sperm cells could be measured, based on the images taken). We chose these two measures because the sperm cells of this species are structurally very simple (basically, a head plus very thin tail). We used mixed effect models (with individual male identity included as a random effect in the model) and model comparison to ask whether males differ in sperm characteristics [66]. Since we collected samples in two years, we also compared models both with and without year (of sample collection) as a fixed effect (2011: *n* = 37, 2012: *n* = 61) in case sperm characteristics differed between years overall and/or in case the magnitude or direction of any differences among male types was moderated by year. We also calculated the median sperm cell tail length and median sperm cell head area for each sample and present these data graphically below. Finally, using mixed effect models and AIC model comparison, we also looked for evidence of a tradeoff between sperm head area and tail length by asking whether there was a significant linear relationship between these variables and whether male type had a significant effect on this relationship, and included individual male identity as a random effect.

## 3. Results

### 3.1. Comparing Absolute Testes Weight and Soma Weight among Male Types

We first compared absolute testes weight among the three male types. We tested for and did not find any extreme outliers. Having found significant deviations from normality using a Shapiro−Wilk test (W = 0.980, *p* < 0.001) and a significant deviation from the assumption of homogeneity of variance using a Levene test (F(2,206) = 9.78, *p* < 0.001), we used a Box−Cox transformation (boxcoxfr in R) and then performed ANOVA on the successfully transformed data. We found significant differences among male types in absolute testes weight (see Figure 1A,F(2, 206) = 56.65, *p* < 0.001). Using a Tukey post hoc test, we found significant differences in testes weight between satellite and sneaker males (HSD = 0.375, *p* < 0.001), satellite males and nesting males (HSD = −0.410, *p* < 0.001), but found no significant difference between sneaker and nesting males in absolute testes weight (HSD = −0.0350, *p* = 0.62). In addition, because we had identified significant differences among the male types in variation in testes weight (Levene test above), we also asked whether there were significant differences between types in the variation in testes weight using an ANOVA on the deviation from the mean testes weight for each type. These residuals had to be transformed using a Box−Cox method due to deviations from the assumptions of normality (W = 0.948, *p* < 0.001). We found that satellite males had significantly greater variation in testes weight than either sneaker males (Tukey HSD = 0.024, *p* = 0.019) or nesting males (Tukey HSD = −0.040, *p* < 0.001), which were not significantly different from one another (Tukey HSD = −0.016, *p* = 0.116). In summary, satellite males have significantly larger testes and significantly greater variance in testes weight than either sneaker males or nesting males.

We also compared absolute soma weight (i.e., total weight−gonad weight) among alternative male types. We found one extreme outlier (a large nesting male) that we excluded from the analyses reported below, though including this sample had no effect on the qualitative results. Due to significant deviations from the assumption of normality (W = 0.979, *p* = 0.004) and the assumption of homogeneity of variances (Levene test F(2,209) = 7.03, *p* = 0.001) that could not be resolved by transforming the data, we conducted a Kruskal−Wallis test. As expected based on prior work [50,57], we found significant differences among male types in soma weight (Figure 1B, Kruskal−Wallis *n* = 209, H = 180, *p* < 0.001) with sneaker males being significantly smaller than both satellite males (Dunn’s H = 5.45, *p* < 0.001) and nesting males (Dunn’s H = 13.4, *p* < 0.001), and satellite males being significantly smaller than nesting males (Dunn’s H = 6.70, *p* < 0.001). Alternative male types also differed in condition factor (soma weight/(standard length)^3^, Kruskal−Wallis test: *n* = 209, df = 2, H = 180, *p* < 0.001); all three types differed significantly from one another with sneaker males having lower condition than either satellites (Dunn’s H = 4.01, *p* < 0.001), or nesting males (Dunn’s H = 11.5, *p* < 0.001), and nesting males having higher condition than satellite males (Dunn’s H = 6.43, *p* < 0.001). Finally, we calculated the gonadosomatic index (gonad weight/total body weight) and showed them graphically (Figure 1C) but used allometric analyses (see below) to compare relative gonad weight among types, statistically. In summary, while satellite males are intermediate in both body size and condition, they have significantly larger testes and greater variance in testes size than either sneaker males or nesting males, despite their cooperative interactions with the nesting male.

### 3.2. Comparing Relative Testes Weight among Male Types

**Figure 2:**
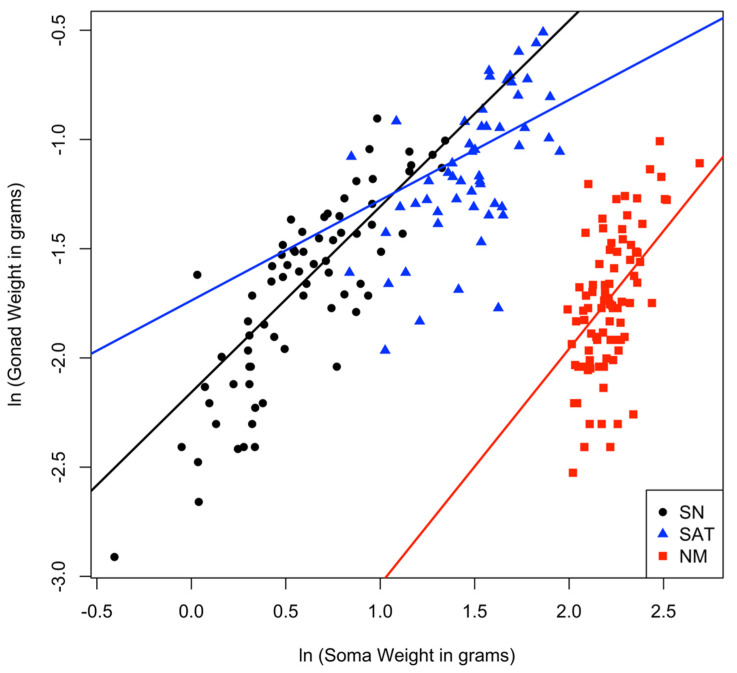
The allometric relationship between soma weight and testes weight differs significantly among alternative male types, based on the mixed effects model reported in Table 1 which fit ln(gonad weight)=intercept + slope ln(soma weight). In this relationship, all three types have significantly different intercepts, and satellite males also have a significantly different slope (such that their gonad weight increases more slowly with soma weight than for the other two male types). Nesting males have a significantly lower intercept than both sneaker and satellite males, consistent with a lower relative investment in gonads. On the legend: SN=sneaker males, SAT=satellite males, NM=nesting males. The allometric relationships (based on the mixed effects model in Table 1) are as follows: sneakers: ln(gonad weight) = 0.85*ln(soma weight)−2.16; satellites: ln(gonad weight) = 0.46*ln(soma weight)−1.74; nesting males: ln(gonad weight) = 1.08*ln(soma weight)−4.11.

In order to ask whether male types differ in relative gonadal allocation, we examined the allometric relationship between testes weight and soma weight, and asked whether this relationship differed significantly among alternative male types. The relationship between the natural log (ln) of testes weight and the natural log (ln) of soma weight appeared to be linear both within and among male types based on visual inspection, and there was no evidence of a deviation from normality of the residuals. There was, however, evidence of a deviation from the assumption of a homogeneity of regression slopes (as detected by a significant interaction between male type and soma weight, F(2, 203) = 3.856, *p* = 0.023 using anova_test(ln(gonad weight) ~ male type × ln(soma weight) in R). We therefore used multilevel linear models to explore this allometric relationship. The best fit model was a mixed effects model that included year as a random effect, assumed random intercepts and slopes, and included an interaction between male type and ln(soma weight). This model found a significant relationship between gonad weight and soma weight as well as significant differences among male types in this relationship (Table 1, Figure 2). The intercept relating ln(gonad weight) and ln(soma weight) differed significantly among all male types. Furthermore, the slope of this relationship differed between sneaker males and satellite males, but was not significantly different between sneakers and nesting males (Table 1, Figure 2, where ln(testes weight) = intercept + slope × ln(soma weight) and therefore testes weight = intercept(soma weight)^slope^). Nesting males have relatively smaller testes for their body size than the other male types overall (i.e., the intercept of their allometric relationship is lower). For satellite males, their testes weight increases at a lower rate with increasing soma weight than the other two male types, though the intercept of that relationship is significantly higher than for sneaker or nesting males (Table 1, Figure 2). Satellite males have larger testes in terms of absolute weight (Figure 1) and a greater relative investment in testes (when controlling for soma weight) than nesting males (Figure 2) even though their rate of investment does not increase at the same rate with body size as sneaker males.

### 3.3. Ejaculate Production and Regeneration

#### 3.3.1. Initial Ejaculate Production

**Figure 3:**
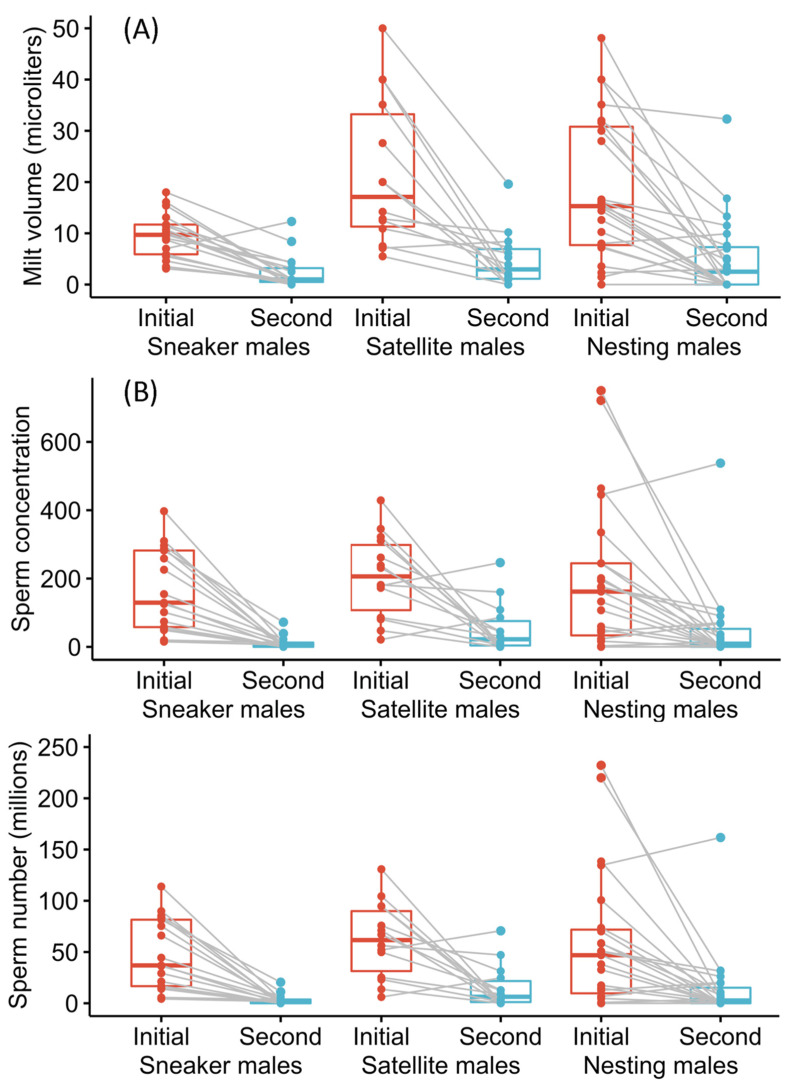
Ejaculate (sperm and seminal fluid) production differs among male types. Initial ejaculate volume (in μL) differed among alternative male types (**A**), while sperm concentration (mean number of sperm cells per microscope image) (**B**) and the total number of sperm cells estimated to be in the entire sample (in millions) (**C**) did not differ among alternative male types. Ejaculate volume, sperm concentration, and sperm number was greater in the initial samples than the second (5 h later) ejaculate samples for all male types. Male types did not, however, differ in sperm or seminal fluid regeneration (i.e., the difference between first and second milt samples). Sneaker male initial ejaculate samples were significantly smaller than satellite or nesting male samples (based on ANOVA followed by Tukey post hoc comparisons). For all three variables and male types, the change between first and second samples was significantly greater than zero (i.e., first sample−second sample). See text for full statistical results.

To ask whether the alternative male types differ in sperm cell and seminal fluid production, we compared the volume, sperm cell concentration and estimated number of sperm (based on volume and concentration) among the three male types for the initial ejaculate (sperm and seminal fluid) samples (Figure 3, *n* = 20 sneakers, 17 satellites and 23 nesting males).

When comparing ejaculate volume among male types, we found a significant deviation from normality (W = 0.939 *p* = 0.005) and a significant nonhomogeneity of variances (F(2,57) = 5.65 *p* = 0.006) that could not be addressed with a Box−Cox transformation. We therefore used a Kruskal−Wallis test and found a significant difference among male types in ejaculate (sperm and seminal fluid) volume (Figure 3, H = 7.41, *n* = 60, df = 2, *p* = 0.025). Sneaker males had significantly smaller volume samples than both satellite males (Dunn’s statistic = 2.49, *p* = 0.013) and nesting males (Dunn’s statistic = 2.20, *p* = 0.028). Satellites and nesting males sample volumes did not differ significantly (Dunn’s statistic = −0.464, *p* = 0.643). Given the significant deviation from the assumption of homogeneity of variance (reported above), we also found (by calculating the residuals and comparing them among types using an ANOVA) that there was a difference among male types in sample volume variation (F(2,57) = 11.64, *p* < 0.001). Sneaker males have significantly lower variation in ejaculate volume than either satellite (Tukey HSD = −7.77, *p* < 0.001) or nesting males (Tukey HSD = 8.43, *p* < 0.001), who did not differ significantly from one another (Tukey HSD = 0.667, *p* = 0. 0.939). Sneakers therefore produce smaller and less variable ejaculate (sperm and seminal fluid) volumes.

When comparing initial sperm cell concentration among male types, we found a significant deviation from normality (W = 0.894, *p* < 0.001), no evidence of significant nonhomogeneity of variances (Levene test F(2,57) = 1.37, *p* = 0.264) and performed a Box−Cox transformation on the data which was able to address the deviation from normality. We found no significant difference in mean sperm counts among male types (ANOVA on transformed data, F(2,57) = 0.382 *p* = 0.68).

For the total estimated number of sperm in the initial sample, we also found a significant deviation from normality (W = 0.886 *p* < 0.001) and but no significant nonhomogeneity of variances (F(2,57) = 1.44, *p* = 0.246). We therefore used a Box−Cox transformation and performed an ANOVA on the transformed data (which met the assumptions of normality and homogeneity of the variances) and found no significant difference among types in the estimated number of sperm (F(2,57) = 0.427, *p* = 0.655).

While we did not find any significant difference among male types in either sperm concentration or the estimated total number of sperm, we did find that sneakers produce smaller ejaculates and had lower variation in ejaculate production than either satellite or nesting males. As sperm number is estimated based on sperm concentration and sample volume, this result is surprising and discussed below. It is worth noting that ejaculate volume was measured in microliters and then diluted in 0.5 mL (500 μL) seawater for sperm cell preservation and counting. This dilution may have made it harder to detect differences in initial sperm cell concentration in the milt. Nonetheless, it is clear that sneakers produced smaller ejaculates. To examine whether evidence of a tradeoff exists, we also asked whether a significant negative statistical relationship existed between ejaculate volume and sperm concentration. We found no evidence of a negative relationship between these variables. Instead, sperm cell concentration actually increased significantly with ejaculate volume and the best fit linear model did not include male type (sperm cell concentration= slope(ejaculate volume)+ intercept, slope = 8.82, t = 6.67, *p* < 0.001; intercept = 36.03, t = 1.36, *p* = 018, Adjusted r^2^ = 0.424).

#### 3.3.2. Ejaculate Regeneration

To answer whether alternative male types differed in the rate at which they regenerate sperm and seminal fluid, we compared differences in sample volume, sperm cell concentration, and the estimated number of sperm between an individual male’s first and second ejaculate (Figure 3B, *n* = 17 sneakers, 14 satellites and 23 nesting males). As above, we checked for outliers and tested for normality and homogeneity of variances and found significant deviations for all three variables. We therefore performed Kruskal−Wallis tests on the difference between first and second samples (comparing these differences among male types). There was a significant decrease in all three variables; the 95% confidence interval around the difference in sample volume, sperm concentration and sperm number between samples did not overlap zero. There was, however, no significant difference among male types and no significant interaction between sample number and male type (change in sample volume: H = 3.14, *n* = 54, df = 2, *p* = 0.209, change in sperm concentration: H = 1.06, *n* = 54, df = 2, *p* = 0.587, change in sperm number: H = 0.920, *n* = 54, df = 2, *p* = 0.631). In summary, while sperm and seminal fluid regeneration was incomplete after 5 h, alternative male types did not differ in their rate of ejaculate regeneration.

### 3.4. Sperm Morphology

**Figure 4:**
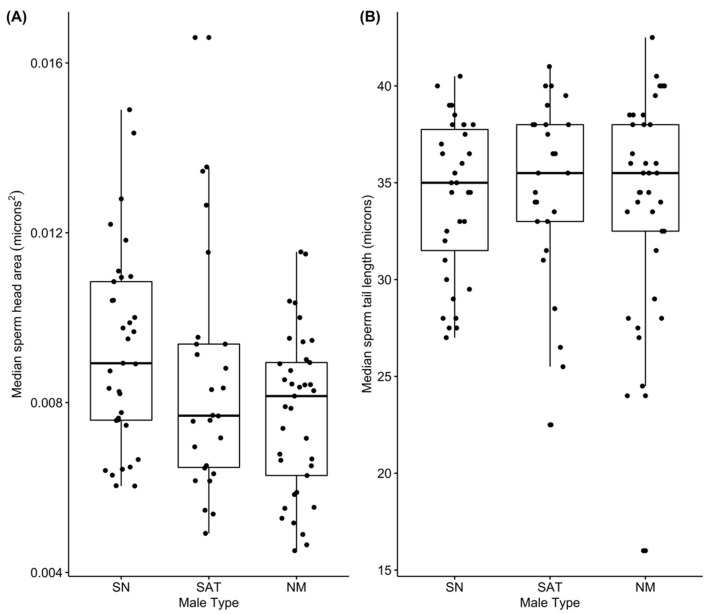
Sperm cell heads (**A**) were significantly larger for sneaker males than either satellite or nesting males. No differences in sperm tail length (**B**) among male types were found. On the x-axis: SN = sneaker males, SAT = satellite males, NM = nesting males. Lower case letters represent the male types that are significantly different from one another. See Table 2 and the text for full details and statistical results.

We asked whether the three male types differed significantly in either sperm tail length or sperm cell head area and whether year of sample collection had any effect on these measures or differences among types (Figure 4). We used mixed effects models (using lme from the nlme package in R) on the raw data for both tail length and head area (i.e., measurement from 10 different sperm cells from each male) to ask whether the sperm cell difference among alternative male types differed in general and between years. We compared three models: (1) with only male type, (2) with male type and year, and (3) with male type, year and an interaction between male type and year. For both variables, the best fit model included only male type. These analyses found significant differences in sperm cell head area among all three males types and no significant differences among male types in sperm cell tail length (Table 2). Sneaker males had the largest sperm cell heads and satellite males had intermediate values with nesting males having the smallest sperm cell heads. We also found no evidence of a tradeoff (or any other significant relationship between) sperm cell head area and tail length (based on AIC of mixed effect models predicting sperm cell area as a function of tail length). In summary, we found no evidence that sperm from alternative male types differed in tail length and we found consistent evidence that sneaker male sperm have the largest heads. While median sperm head size was lower in satellite males (Figure 4A), the mixed effect model found that nesting male sperm have significantly smaller heads than either sneaker or satellite males (Table 2).

## 4. Discussion

Extensive research has examined how and why the allocation to and characteristics of sperm and ejaculates differ between species and among alternative male types within species. Yet, significant unexplained variation remains [7,11,23]. Here we asked how divergent social roles in general, and the presence of cooperation among unrelated males to reduce sperm competition in particular, affect how much and what kind of sperm and ejaculate males produce. In the ocellated wrasse, we found differences among alternative male types (that differ in their social roles and interactions) in absolute testes size, the allometric relationship between testes and body size, ejaculate production, and sperm cell morphology. In particular, we found that satellite males, which cooperate behaviorally with nesting males to reduce sperm competition from sneaker males, have the largest absolute testes (Figure 1), exhibit a relative investment in testes that is similar to purely competitive sneaker males (Figure 2), and have equally large ejaculates (Figure 3) and a competitive sperm cell morphology (Figure 4) as compared to their nesting male partners. These patterns differ significantly from what one might expect based on their behaviorally cooperative role. Yet, they also differ from the patterns predicted based solely on differences among male types in sperm competition. Our findings, therefore, suggest that considering the presence of, and synergy between, cooperation and competition among males, will increase our ability to explain the observed patterns of sperm and ejaculate production in this and other species.

To explore these patterns further, it is informative to compare the patterns reported here with results from other species in which alternative male types occur. For example, a very similar pattern of testes allometry was found in bluegill sunfish, even though there is no reproductive cooperation among males in this species. In the bluegill, the parental males had significantly greater slopes relating ln(testes mass) to ln (soma mass) than either sneaker or noncooperative satellite males (which behave as female mimics) [67]. In contrast to the ocellated wrasse, sperm cell concentration was higher in bluegill sneaker and (noncooperative) satellite males than nesting males [67], as expected, based on the differences in sperm competition these male types experience.

Nesting males in the ocellated wrasse had significantly lower relative investment in testes, consistent with theoretical expectations, as well as documented allocation patterns in other fish mating systems in which only (noncooperative) nesting and sneaker males coexist (e.g., [68]). Our results are also consistent with the pattern of testes size found among different male morphs in ruffs, in which cooperative satellite males have larger testes than independent (dominant) males [69].

In two species of cichlid in which subordinate males help dominant males defend their territory and provide care, these subordinate (helper) males had much smaller testes and lower sperm production then the dominant, parental males [25,37,39]. In these species, subordinate males also have relatively few mating opportunities and low rates of paternity, which may also contribute to their low investment in testes and sperm production [70,71]. By comparison, while satellite males in the ocellated wrasse are intermediate in both body size and condition, they had significantly larger absolute testes weights than either sneaker males or nesting males, despite their cooperative interactions with the nesting male. It is worth noting that cooperation plays a very different role in this species; satellite males chase away sneaker males to reduce sperm competition for the nesting male and in return are allowed closer to the nest and greater mating opportunities [41,51].

These results also suggest that the evolution of the cooperative interactions among satellite and territorial males could be explained in part by the ability of these subordinate males to behave cooperatively, while also investing in sperm production, presumably increasing their fitness advantage in sperm competition. Given that satellite males have significantly greater variance in testes weight, individual satellite males also differ in the risk and intensity of sperm competition they represent to their nesting male “partners”. This greater variation in testes weight among satellite males may be explained by the fact that satellites are a mix of one or two year old males at our study site, while all sneakers are one year old and all nesting males are two years old [52]. However, it is also worth noting that satellite males are more variable in testes weight but not in body size or condition, indicating age and size may not be the only factors contributing to this variation in gonadal allocation. In addition, sneakers and satellite males differ significantly in their relative investment in testes, with the satellite male investment being higher (i.e., a significantly greater intercept) but increasing at a lower rate with body size than in sneaker males (i.e., significantly lower slope). This could be a concession to their nesting male “partners” (i.e., that large satellite males invest relatively less in testes weight for their size). However, the interaction between male type and soma weight makes direct comparison of relative investment difficult given that male types also differ in body size. Variation among satellite males in allocation to testes warrants further study, especially given their cooperative and competitive role in sperm competition.

Surprisingly, however, we found no differences in sperm production or regeneration among male types, though sneaker male sperm and seminal fluid samples were of significantly smaller volume, consistent with sneaker males producing less seminal fluid. The testes of the ocellated wrasse do not have any obvious accessory gland structures (which are rare in fishes and have to our knowledge not been reported in this group of fishes, i.e., the Labrines or family Labridae). For comparison, in midshipman, parental guarding males invest more in accessory glands and related structures that produce seminal fluid, and the seminal fluid they produce increases the velocity of guarder male sperm relative to (noncooperative) sneaker male types [28,29]. Sneaker male ejaculates in midshipman (which experience higher sperm competition than parental males) have more sperm but less seminal fluid than guarding males. Furthermore, in grass gobies, alternative male types differ significantly in seminal fluid production [72]. In addition, the sperm of sneaker males was positively affected by the nesting male seminal fluid, while sneaker male seminal fluid had a negative effect on the sperm of nesting males [72]. It is therefore especially interesting that cooperative satellite males in the ocellated wrasse (which release less sperm per spawning event than sneaker males [56]) produce the same volume of milt as nesting males (as reported here). It remains puzzling, however, that male types differed in testes size, but not in the estimated number of sperm cells produced. Further research on sperm and seminal fluid production and regeneration is needed to fully understand how and why alternative male types differ in both seminal fluid and sperm production in the ocellated wrasse and what role the cooperative interactions between satellite and nesting males plays in driving these patterns. For example, further studies should examine the production and function of seminal fluid as well as the condition-dependence of sperm and seminal fluid production within and among male types. Furthermore, our analyses found no evidence of tradeoffs between sperm and seminal fluid production, though further research considering differences in the energy available for reproduction are needed.

Finally, we found that sneaker males produced sperm cells with larger heads. Sperm tail length did not differ among male types despite the increased risk and intensity of sperm competition experienced by sneaker and satellite males, relative to the nesting male. This difference in sneaker male sperm morphology is consistent with prior research showing that sneaker males produce sperm with lower velocity and motility [63] and sneaker male testes have a significantly demasculinized gene expression compared to satellite and nesting males [55]. Although there has been extensive research on how male alternative reproductive types differ in sperm characteristics [23], this comparison is to our knowledge the first to do so in a species in which cooperative interactions among alternative males types serve to reduce sperm competition and increase mating opportunities for the cooperative partners. Our results indicate that social roles in general, and the presence of cooperation with respect to mating in particular, may influence male sperm characteristics in important ways. However, it is not as simple as behaviorally cooperative males (e.g., satellite males) producing less competitive sperm or ejaculates. Instead, here we found that the most behaviorally cooperative males actually had not only the largest testes, but also larger ejaculates and sperm traits associated with greater velocity, than the purely competitive sneaker males.

Further study is also needed to understand how male condition, energy budgets and the resulting tradeoffs between different components of fitness affect the observed patterns of sperm production and ejaculate characteristics in general. We have few predictions for how alternative male types should differ in sperm characteristics or how those sperm traits are expected to covary with ejaculate allocation, especially in cases where the male alternative male types are plastic and depend on male size, age or condition (see [23] for further discussion). In addition, current theory has not considered the possibility for behavioral cooperation among males and how this is predicted to affect ejaculate allocation or characteristics. Yet, positive interactions among sperm are known to occur [43] and can be favored under sperm competition. In addition, theoretical and empirical work has shown that sperm allocation and ejaculate characteristics depend in important ways on the interplay between cooperation and conflict between the sexes over mating and fertilization [73,74,75,76,77]. We suggest that our ability to predict and explain the observed patterns of sperm evolution and ejaculate production will increase if we consider not only competition among males, but also how cooperation among males and social interactions generally affect the evolution and expression of postcopulatory sexual selection and the traits that arise in response to selection arising from variation in fertilization success. Future research should also consider how social and gametic interactions within and between the sexes simultaneously shape the many varied aspects of sperm and seminal fluid production.

## 5. Conclusions

Despite extensive theoretical and empirical research on ejaculate allocation and sperm traits [1,2,3,4,5,7,8,9,77,78,79], extensive unexplained variation in these traits remains. We argue that further study of male social roles and interactions may help explain some of this variation, especially in species with alternative male types that differ not only in sperm competition but also in their social roles and energy budgets. Despite their behaviorally cooperative role, satellite males in this species have the highest absolute investment in testes, higher relative investment in testes than nesting males, and produce ejaculates that are equivalent to their dominant nesting male partners. Further theoretical and empirical research predicting the patterns of sperm and seminal fluid production is needed that explicitly considers not only the risk and intensity of sperm competition, but also how males interact behaviorally with one another during reproduction, and how these behavioral interactions coevolve with sperm and ejaculate traits.

## Figures and Tables

**Table 1:** The allometric relationship between soma weight and testes weight based on the mixed effects model: ln(gonad weight) = male type × ln(soma weight) fit using maximum likelihood. The model included year as a random effect, assumed random intercepts and slopes, and included an interaction between male type and ln(soma weight). The parameters below are based on fitting the model using sneaker males as the baseline. The model was fit using lme in the nmle package in R. Variables and interactions with significant effects are in bold. Degrees of freedom = 197, *n* = 209.

Variable	Estimate	Standard Error	*t*-Value	*p*-Value
**Intercept**	−2.16	0.08	−28.4	<0.001
**Male type: satellite**	0.42	0.21	1.98	0.0488
**Male type: nesting**	−1.96	0.46	−4.28	<0.001
**ln(soma weight)**	0.85	0.08	10.53	<0.001
**ln(soma weight) × satellite**	−0.39	0.15	−2.57	0.0108
**ln(soma weight) × nesting**	0.23	0.22	1.05	0.2930

**Table 2:** Results of the best fit mixed effect models comparing sperm cell head area and tail length among alternative male types with the sneaker male type as the baseline. Variables and interactions with significant effects are in bold.

	Variable	Estimate	Standard Error	df	*t*-Value	Prob > |t|
Sperm Cell Head Area (mm^2^)	**Intercept**	0.000009529	3.939 × 10^−7^	42.77	24.193	<0.001
	Type: satellite	−0.000004820	6.037 × 10^−7^	42.82	−0.798	0.429
	**Type: nesting**	−0.000001770	5.495 × 10^−7^	42.77	−3.221	0.002
Sperm Tail Length (mm)	**Intercept**	0.03442	0.00113	97.33	30.232	<0.001
	Type: satellite	0.00128	0.00175	97.89	0.731	0.467
	Type: nesting	−0.00084	0.00159	97.33	−0.527	0.600

## Data Availability

The data presented in this study will be made openly available in Dryad, once the paper has been accepted for publication.
